# Supplementary material for: Inhibitory Mechanism of Trichoderma virens ZT05 on Rhizoctonia solani
Source: Plants (Basel). 2020 Jul 19;9(7):912. doi: 10.3390/plants9070912 (PMC7412022; doi:10.3390/plants9070912)
Supplement: Supplementary file 1 [file plants-09-00912-s001.pdf]

Supplementary Table S1

Gene name	Log2FC (Rs_Tv/Tv)	Padjust	Length	Swiss-prot-hit-name	Swiss-prot description
**Extracellular Proteases**					
TRIVIDRAFT_81735	1.871359288	1.36495E-13	3326	sp|Q7SA85|LONP2_NEUCR;	Lon protease homolog 2, peroxisomal
TRIVIDRAFT_63956	2.422062731	1.14807E-12	1569	sp|E3QWD3|NPIIA_COLGM;	Neutral protease 2 homolog MGG_10927
**Oligopeptide transporters**					
TRIVIDRAFT_58191	1.631203	8.44E-09	2740	sp|O82485|OPT7_ARATH;	Oligopeptide transporter 7
**G-protein coupled receptors**					
TRIVIDRAFT_35938	1.212964	4.02E-10	20134	sp|Q99144|PEX5_YARLI;	Peroxisomal targeting signal receptor
TRIVIDRAFT_30459	1.033583	0.000915	1064	sp|Q09749|ADRL_SCHPO;	ADIPOR-like receptor SPBC12C2.09c
TRIVIDRAFT_59177	1.166872	0.014158	3775	sp|Q8BTN6|LENG9_MOUSE;	Leukocyte receptor cluster member 9
TRIVIDRAFT_138511	1.39178	0.040013	576	sp|P57078|RIPK4_HUMAN;	Receptor-interacting serine/threonine-protein kinase 4

Supplementary Table S2

Gene name	Log2FC (Rs_Tv/Tv)	Padjust	Length	Swiss-prot-hit-name	Swiss-prot description
**Chitinase**					
TRIVIDRAFT_178019	2.615454	4.65E-13	1335	sp|Q12713|CHI33_TRIHA;	Endochitinase 33
TRIVIDRAFT_89999	1.658827	5.41E-10	1371	sp|Q8NJQ5|CHI37_TRIHA;	Endochitinase 37
ECH1	1.39482	2.4E-06	1632	sp|A6N6J0|CHI46_TRIHA;	Endochitinase 46
TRIVIDRAFT_69839	1.110896	0.000115	3198	sp|P32470|CHI1_APHAL;	Chitinase 1
TRIVIDRAFT_213202	2.339033	0.000256	1596	sp|E9ENC6|CHI3_METRA;	Endochitinase 3
CHT1.1	1.453396	0.000675	1469	sp|Q12713|CHI33_TRIHA;	Endochitinase 33
**Glucanase**					
TRIVIDRAFT_89797	1.615731	1.63E-08	1893	sp|A0A024SH20|GUN2_HYPJR;	Endoglucanase EG-II
TRIVIDRAFT_72072	2.02365	1.7E-07	1565	sp|P23548|GUN_PAEPO;	Endoglucanase
TRIVIDRAFT_27891	1.873419	2.41284E-06	1179	sp|Q0CEU4|EGLD_ASPTN;	Probable endo-beta-1,4-glucanase D
TRIVIDRAFT_42536	1.805727	0.006974728	834	sp|P22669|GUN_ASPAC;	Endoglucanase-1
TRIVIDRAFT_28149	1.834324	0.00858733	849	sp|A1D4F1|XGEA_NEOFI;	Probable xyloglucan-specific endo-beta-1,4-glucanase A
TRIVIDRAFT_76895	1.171856	0.011841965	1655	sp|Q7M4T0|NEG1_NEUCR;	Endo-1,6-beta-D-glucanase
**Protease(proteasome)**					
TRIVIDRAFT_176639	1.08760	0.0013236	1450	sp|E9ES90|STS1_METRA;	Tethering factor for nuclear proteasome STS1

Supplementary Table S3

Gene name	Log2FC (Rs_Tv/Tv)	Padjust	Length	Swiss-prot-hit-name	Swiss-prot description
**Tetracycline resistance proteins**					
TRIVIDRAFT_57595	1.326570	0.000512	3735	sp|Q01911|TETX_BACFG;	Tetracycline resistance protein from transposon Tn4351/Tn4400
TRIVIDRAFT_219995	1.458342	0.010485	1140	sp|E9R876|GLIA_ASPFU;	MFS gliotoxin efflux transporter gliA
**Reductase**					
TRIVIDRAFT_62654	1.294983	0.046806	1084	sp|P39315|QOR2_ECOLI;	Quinone oxidoreductase 2
TRIVIDRAFT_211837	1.793853	3.275094	1619	sp|A7RK30|QORL2_NEMVE;	Quinone oxidoreductase-like protein 2 homolog
TRIVIDRAFT_38645	2.264700	0.005178	789	sp|A0A084R1K2|ATR9_STAC4;	Short-chain dehydrogenase/reductase ATR9
TRIVIDRAFT_42391	1.415816	0.000231	972	sp|Q53FA7|QORX_HUMAN;	Quinone oxidoreductase PIG3
TRIVIDRAFT_45041	1.021142	0.000158	762	sp|A0A084R1I4|ATR10_STAC4;	Short-chain dehydrogenase/reductase ATR10
TRIVIDRAFT_50977	2.939743	1.383962	3653	sp|Q9Y8G7|C505_FUSOX;	Bifunctional cytochrome P450/NADPH--P450 reductase
TRIVIDRAFT_68923	2.929785	7.664841	5558	sp|Q9Y8G7|C505_FUSOX;	Bifunctional cytochrome P450/NADPH--P450 reductase
TRIVIDRAFT_69465	2.091020	4.233595	1211	sp|Q53FA7|QORX_HUMAN;	Quinone oxidoreductase PIG3
TRIVIDRAFT_71556	1.026713	0.000117	2783	sp|A2QS05|NCPR_ASPNC;	NADPH--cytochrome P450 reductase
**The heat shock response**					
TRIVIDRAFT_216898	1.548984	0.000001	3182	sp|P31540|HSP98_NEUCR;	Heat shock protein hsp98
TRIVIDRAFT_80583	2.087637	0.000001	2645	sp|O74402|HSP78_SCHPO;	Heat shock protein 78, mitochondrial
TRIVIDRAFT_215292	2.040524	0.000008	1032	sp|P19752|HSP30_NEUCR;	30 kDa heat shock protein
TRIVIDRAFT_210885	1.331958	0.000009	2378	sp|Q01233|HSP70_NEUCR;	Heat shock 70 kDa protein
TRIVIDRAFT_89650	1.180429	0.000049	2885	sp|O43109|HSP90_PODAS;	Heat shock protein 90 homolog
TRIVIDRAFT_195722	1.697725	0.000104	3374	sp|Q9CZJ2|HS12B_MOUSE;	Heat shock 70 kDa protein 12B
TRIVIDRAFT_78895	1.313931	0.000416	2608	sp|Q5B0C0|HSP7M_EMENI;	Heat shock 70 kDa protein
TRIVIDRAFT_217094	1.707831	0.000769	1016	sp|P19752|HSP30_NEUCR;	30 kDa heat shock protein
**The oxidative stress response**					
TRIVIDRAFT_207997	3.32	0.000004	1385	sp|Q9SIE0|ALKB2_ARAT	DNA oxidative demethylase

				H;	ALKBH2
**ABC efflux transporters**					
TRIVIDRAFT_33722	3.058718	1.000032	6901	sp|Q54U44|ABCCC_DICDI;	ABC transporter C family member 12
TRIVIDRAFT_52608	2.828204	6.685599	5033	sp|P53756|PDR18_YEAST;	ABC transporter ATP-binding protein/permease PDR18
TRIVIDRAFT_86623	1.904562	3.892920	5338	sp|I1RL06|ZRA1_GIBZE;	ZEB2-regulated ABC transporter 1
TRIVIDRAFT_36031	2.62432	3.623095	5676	sp|Q8J2Q1|FUM19_GIBM7;	ABC transporter FUM19
TRIVIDRAFT_85589	2.208254	6.399924	4840	sp|I1RL06|ZRA1_GIBZE;	ZEB2-regulated ABC transporter 1
TRIVIDRAFT_190418	1.268807	0.000066	4858	sp|K0E4D9|ECDL_ASPRU;	ABC transporter ecdL
TRIVIDRAFT_45576	1.104975	0.000530	4180	sp|Q08234|YO075_YEAST;	Uncharacterized ABC transporter ATP-binding protein/permease YOL075C
TRIVIDRAFT_83793	1.480492	0.003585	5036	sp|I1RL06|ZRA1_GIBZE;	ZEB2-regulated ABC transporter 1
**Multidrug resistance transporters**					
TRIVIDRAFT_76205	3.472369	1.114803	2237	sp|Q6FV98|TPO12_CANGA;	Multidrug transporter TPO1_2
TRIVIDRAFT_192676	3.777513	1.390707	2219	sp|Q6FV98|TPO12_CANGA;	Multidrug transporter TPO1_2
